# The effects of dementia care mapping on nursing home residents’ quality of life and staff attitudes: design of the quasi-experimental study Leben-QD II

**DOI:** 10.1186/1471-2318-13-53

**Published:** 2013-06-01

**Authors:** Margareta Halek, Martin Nikolaus Dichter, Tina Quasdorf, Christine Riesner, Sabine Bartholomeyczik

**Affiliations:** 1 German Center for Neurodegenerative Diseases (DZNE), Witten, Stockumer Straße 12, 58453, Witten, Germany; 2 School of Nursing Science, Witten/Herdecke University, Stockumer Straße 12, 58453, Witten, Germany

## Abstract

**Background:**

The main objective of care for people with dementia is the maintenance and promotion of quality of life (Qol). Most of the residents in nursing homes have challenging behaviors that strongly affect their Qol. Person-centered care (PCC) is an approach that aims to achieve the best possible Qol and to reduce challenging behaviors. Dementia Care Mapping (DCM) is a method of implementing PCC that has been used in Germany for several years. However, there are no data on the effectiveness of DCM or the challenges of implementation of DCM in German nursing homes.

**Methods/design:**

In this quasi-experimental non-randomized cluster-controlled study, the effects of DCM will be compared to 2 comparison groups. 9 nursing homes will take part: 3 will implement DCM, 3 will implement a comparison intervention using an alternative Qol assessment, and 3 have already implemented DCM. The main effect outcomes are Qol, challenging behaviors, staff attitudes toward dementia, job satisfaction and burnout of caregivers. These outcomes will be measured on 3 data points. Different quantitative and qualitative data sources will be collected through the course of the study to investigate the degree of implementation as well as facilitators of and barriers to the implementation process.

**Discussion:**

This study will provide new information about the effectiveness of DCM and the implementation process of DCM in German nursing homes. The study results will provide important information to guide the national discussion about the improvement of dementia-specific Qol, quality of care in nursing homes and allocation of resources. In addition, the study results will provide information for decision-making and implementation of complex psychosocial interventions such as DCM. The findings will also be important for the design of a subsequent randomized controlled trial (e.g. appropriateness of outcomes and measurements, inclusion criteria for participating nursing homes) and the development of a successful implementation strategy.

**Trial registration:**

Current Controlled Trials ISRCTN43916381.

## Background

The main objective in providing care for people with dementia is maintenance and promotion of quality of life (Qol) [1]. Care of nursing home residents with dementia is a challenge for staff, particularly when those residents exhibit challenging behaviors [2]. The prevalence of challenging behavior in people with dementia is 78% globally [3]. Similarly, [4] up to 90% of German residents with dementia have challenging behaviors [5]. The most frequent symptoms are depressive symptoms followed by aggression [5]. Challenging behaviors strongly affect the well-being of people with dementia [6] and have a negative impact on stress levels and job satisfaction of the staff [2].

Person-centered care (PCC) is described as an approach to achieve the best possible Qol for people with dementia and to reduce challenging behaviors [7].

There is consensus that PCC focuses on the individuality of the person with dementia and not on his or her impairments [8-10]. The theoretical underpinning of PCC is Kitwood’s social-psychological theory of personhood [11]. This theory sets the sense of personal worth, agency, social confidence and hope as global states of well-being for persons with and without dementia. The physical and social environment influence wellbeing and behavior of persons with dementia in a similar way [12,13]. An appropriate physical environment should provide outdoor space, facilitate orientation, offer different activity areas and enough space to walk around. Social environment factors are e.g. continuity and presence of staff, verbal and nonverbal inclusion of individuals in activities and respectful ways of communication. Internationally, PCC has become an important component of dementia guidelines [14,15] and research activities [7,16-18].

The Dementia Care Mapping (DCM), a structured observational assessment instrument, was developed by Kitwood & Bredin [19] to evaluate and advance the implementation of PCC in institutional and day care settings. The DCM tool was updated by the Bradford Dementia Group to the current 8^th^ version [20]. DCM is a systematic approach for the assessment of PCC that can help to identify factors influencing behavior and to create individual person-centered care plans [16,21]. DCM can also be used as an assessment for residents’ well-being and Qol [22,23]. The DCM observational instrument is embedded in the DCM method, which consists of 6 fixed components: 1) briefing and preparation of care staff and leadership, 2) DCM observation, 3) DCM data analysis and report-writing, 4) feedback of results to care staff and leadership, 5) action-planning by care staff based on the DCM results and 6) realization of the action plan [24].

The engagement of care staff to take an active and accountable role is part of the DCM process. This process is repeated to create an ongoing development of PCC. The successful use of DCM to advance PCC has been reported in several studies [7,16,23,25,26]. Positive effects included improved well-being of residents, higher quality staff interaction [23] and lower rates of agitation [7]. Additionally, DCM use might help staff to adopt a holistic approach when attending to the needs of residents [16]. The effects of DCM are highly dependent on the implementation process, which is influenced by multiple factors [24]. Moreover, the implementation of DCM has to be carefully considered because of relatively high costs in time, staff education, and external consulting for example, [7,8] compared to the implementation of other Qol tools.

PCC and DCM have been equally well known in Germany for several years. The first trainings in DCM were carried out in 1998, and interest has been continuously growing. DCM is used in care homes, small-scale living arrangements and day care settings. Since 2009, the increasing interest in using DCM and other Qol measurements in German nursing homes has partly been emerged from the assessment of residents´ quality of life through care audits of the medical advisory service of the statutory health insurance [27]. Up to now there has been no study about the effectiveness of DCM on residents with dementia and nursing staff in Germany. There are some evaluation reports about DCM projects [28,29] with hypotheses generating results for Germany. These reports suggest that DCM enhances PCC and that a successful implementation depends on several factors.

Results indicate that for a successful use of DCM staff members need to have a good interaction quality within the team, they should have higher job satisfaction and a small rate of staff turnover. If teams have these characteristics, residents wellbeing seems to increase [28].

## Study aim and research question

The aims of this study are to evaluate the effects of DCM in dementia care in German nursing homes, to explore the implementation of DCM and to perform a cost-effectiveness analysis. These aims were translated into the following research questions:

Effects:

1. Does the DCM method positively affect the Qol of people with dementia and reduce their challenging behaviors?

2. Does the DCM method positively affect staff attitudes toward dementia, improve job satisfaction and reduce burnout?

Process evaluation:

3. To what extent could the intervention be implemented (degree of implementation)?

4. What are facilitators of and barriers to DCM implementation?

Cost-effectiveness:

5. Does the DCM method positively affect the efficiency of dementia care compared to other interventions?

## Methods/design

### Design

**Figure 1:**
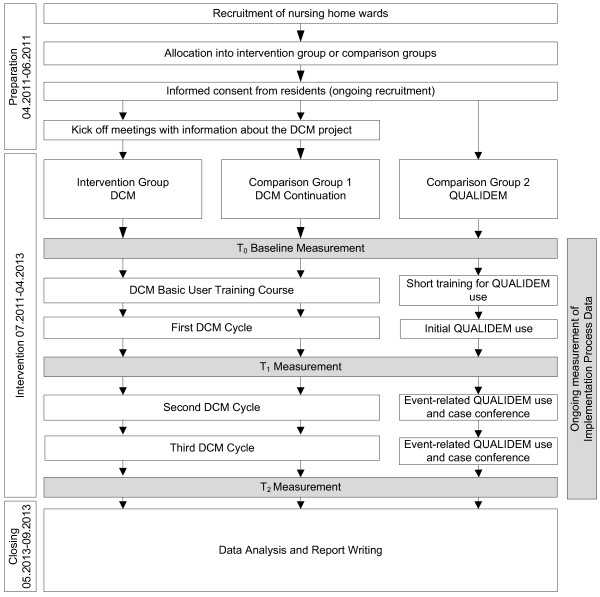
Study design.

To measure the effects of DCM a quasi-experimental design is being used, with 3 measurement points (Figure 1). 9 nursing home units will be assigned to either the DCM intervention group or 1 of 2 comparison groups. The care provider will be responsible for the assignment of a unit to 1 of the 3 groups. The DCM method will be implemented in the 3 units assigned to the intervention group. The first comparison group will consist of 3 nursing units that have at least 3 years of experience in the use of the DCM method. The 3 units in the second comparison group will receive a control intervention based on the use of the dementia-specific Qol-measurement QUALIDEM. Quantitative methods will be used to study the effects and cost-effectiveness. A broad explorative mixed-methods approach will be used for the process evaluation.

### Sample

The target populations are residents with dementia and the care teams of 9 nursing home units are primarily located in North-Rhine Westphalia, Germany. All of the participating nursing homes belong to the same nursing home provider. 1 living unit from each nursing home is participating in the study. After allocation of the units to 1 of the 3 groups, written information about the study will be given to the residents’ legal representatives, and written informed consent will be obtained from them. Inclusion criteria for the residents are Functional Assessment Staging (FAST) [30] score ≥ 2 and a length of stay ≥ 2 weeks on the respective nursing home unit. Based on the high likelihood of attrition, the target population participants who are lost to follow up will be replaced with new participants. All participating caregivers receive written information about the study and are invited to participate at a kick-off information meeting. After that, the voluntary completing the questionnaire is considered as informed consent. From the caregivers who will participate in a interview a written informed consent will be obtained.

### Intervention

For the coordination and management of each intervention group, 1 staff member in each nursing home will be deployed. The interventions for the 3 groups are described in detail.

#### Intervention group

The DCM method consists of some fixed components that will be implemented in the following way:

Briefing and preparation: different kick-off meetings will be held for each of the involved care teams. Staff will meet as a group and then together with management to learn about the DCM project, to answer questions, to reduce concerns and to recruit staff members who might be interested in a DCM role.

1. Staff Training

 A certified DCM basic user training with 6 participants (2 members from each nursing home) will be carried out by the in-house DCM trainer before the intervention starts. DCM mappers will be selected based on interest and will have distinct professional roles from nursing assistants and head nurses. After basic user training all 12 new mappers and the in-house DCM trainer will have the opportunity to improve their skills in 1.5 day coding sessions for each nursing home to practice the DCM observation.

2. DCM Cycle

 The DCM cycle for all 3 units will include an 8-hour DCM observation (except for the first mapping, which will be 5 hours due to less mapping experience), provision of feedback to care staff within 1 week and action-planning within 4 weeks. 2 DCM cycles will be set each year. Mappers of 1 nursing home will always observe together in 1 associate institution and will undertake data analysis and feedback together (cross over). For the first year of the DCM project, the DCM trainer will accompany each mapping-pair during observation, data analysis and feedback. After this period, the mappers will work as pairs without additional assistance. Separate feedback will be given to interested family caregivers beginning in the second year of the project.

#### Comparison group 1

The DCM process has been ongoing since 2009 in the comparison group units. These units received 2 DCM cycles per year. The mapping process before the project Leben-QD II was performed by an external DCM mapper. Therefore, a certified DCM basic user training with 6 participants (2 members of each nursing home) will also be carried out at the beginning of the intervention phase. The selection of the training participants and the opportunity to improve skills in additional coding sessions will be equal to the procedures used in the intervention group. This applies also to all other intervention components mentioned above. We assume that there will be a greater benefit for residents and caregivers in the intervention group where DCM was implemented before the start of the study because this group has caregivers that are more experienced with PCC. Furthermore, it will be possible to investigate the facilitators of and barriers to DCM implementation using a long-term perspective.

#### Comparison group 2

Based on the quality assurance criteria of the German medical advisory service of the statutory health insurance about the well-being of people with dementia and the integration of study results into the care process, it was not possible designate 1 group to care as usual. The dementia-specific measurement QUALIDEM [31] was selected as a comparison intervention because it is known in Germany and, therefore, is a feasible option for assessing Qol in nursing homes. The assumption is that simply measuring Qol leads to a greater awareness of Qol by caregivers and supports the discussion on how to maintain or enhance it.

Compared to the DCM, the QUALIDEM has no fixed implementation steps, and completing of the QUALIDEM instrument takes less time than does observation with DCM. QUALIDEM requires less staff training and is free of charge.

For comparison group 2, the use of the QUALIDEM as a Qol assessment tool is nested in the following intervention components:

1. A short (1.5 hours) training program for staff on the 3 units, including some general information about Qol assessment for people with dementia and the usage of QUALIDEM in particular.

2. QUALIDEM usage as follows

 An initial Qol-rating will be given to all residents with dementia within 3 months of the training (October – December 2012). An additional Qol assessment will be conducted if notable changes in the residents’ needs are perceived. At least every 6 months, a new Qol assessment must be carried out. The Qol assessments will always be based on the proxy-rating agreements of 2 staff members (nurses or social workers) in relation to a retrospective observation period of 2 weeks. If necessary, staff will initiate case conferences based on the results of the Qol ratings, to understand the individual needs of a particular resident. The care team will participate in these case conferences.

## Measures/data collection

To study the intervention effects, quantitative measurements will be used to measure resident and staff variables. The process evaluation will use quantitative data from residents, staff and organizational variables as well as data from the process documentation and staff interviews and observations (Table 1).

**Table 1:** Measurement instruments

**Residents**		
**Variable**	**Instrument/source**	**Type of variable**
Demographic variables	Single items	Control variables
Qol	Quality of Life-Alzheimer’s Disease (Qol-AD)	Primary outcome
Qol	QUALIDEM	Secondary outcome
Neuropsychiatric symptoms	Neuropsychiatric Inventory – Nursing Homes (NPI-NH)	Secondary outcome
Dependency	Physical Self-Maintenance Scale (PSMS)	Control variables
Dementia severity	Functional Assessment Staging (FAST)	Control variable
Medication	Medication documentation sheets	Control variable
**Staff**		
**Variable**	**Instrument/source**	**Type of variable**
Demographic variables	Single items	Control variables
Perception of dementia	Attitudes to Dementia Questionnaire (ADQ)	Primary outcome
Job satisfaction	Copenhagen Psychosocial Questionnaire (COPSOQ) (4 Item Scale)	Secondary outcome
Burnout	Copenhagen Burnout Inventory (CBI)	Secondary outcome
Dementia-specific burden	BelaDem Questionnaire	Control variables
**Implementation process**		
**Domain**	**Instrument/source**	**Type of variable**
Intervention characteristics outer setting, inner setting, characteristics of individuals process of implementation	Interviews with head nurses and staff members	Influencing factor
Characteristics of individuals	single items of staff questionnaire	Influencing factor
Inner setting	DIQ questionnaire	Influencing factor
Inner setting	SYMLOG	Influencing factor
Process of implementation	Process documents (for example, posters, protocols, mail)	Influencing factor
Fidelity of implementation	Interviews with head nurses and staff members; single items of staff questionnaire; process documents (posters, protocols, action plans)	Degree of implementation
Successful implementation of PCC	Resident records	Degree of implementation
Environmental changes	Dementia Milieu Assessment (DMA)	Degree of implementation
**Cost effectiveness**		
**Domain**	**Instrument/source**	**Type of variable**
Resource use	Nursing home administration system	Cost outcome

The data collection times will be oriented with the DCM cycles in the intervention group and will take place before starting the implementation (DCM training) (T0, months 1 to 3) and no more than 2 months after the first (T1, month 9–11) and the third (T2, months 21–23) DCM-cycle. The data collection times for the process evaluation and the implementation process will correspond with T0 to T2 (Figure 1). Cost data for the economic evaluation will be extracted from the nursing home administrative system at the end of the intervention period.

### Effect evaluation

Measurement instruments were chosen based on their appropriateness for the target setting and population, psychometric properties (validity and reliability) and their feasibility. Table 1 summarizes all measurement instruments.

#### Measures for people with dementia

The primary outcome, Qol of residents with dementia, will be measured with the Quality of Life-Alzheimer’s Disease (Qol-AD) proxy-version [32]. Qol-AD is a 13-item scale that measures the Qol domains including physical condition, mood, memory, functional abilities, interpersonal relationships, ability to participate in meaningful activities, financial situation and global assessment of self as a whole and Qol as a whole. Sum scores range from 13 to 52, higher scores indicate a greater Qol. The Qol-AD is a questionnaire that is completed by caregivers. The internal consistency, inter-rater reliability and construct validity of the proxy version showed acceptable results [32].

Secondary outcome measures include the QUALIDEM [31] and the Neuropsychiatric Inventory – Nursing Homes (NPI-NH) [33]. The QUALIDEM was chosen as a secondary outcome because it is the only Qol instrument with a strong focus on care of relevant psychosocial aspects of Qol [31]. This proxy-measurement for Qol of people with mild to severe dementia is assessed with a 37-item version covering 9 domains of Qol: the care relationship, positive affect, negative affect, restless or tense behavior, positive self-image, social relationships, social isolation, feeling at home and having something to do. The domains of positive self-image, feeling at home and having something to do cannot be assessed in people with very severe dementia. Thus, the second version is made up of 18 items covering 6 domains of Qol. Higher scores on Qol subscales indicate better Qol. QUALIDEM was developed and validated between 2005 and 2007 by Dutch researchers (Ettema et al. 2007). In 2008, QUALIDEM was translated into German. In an initial investigation, the German version was deemed to be valid based on factor analysis and moderate to high internal consistency [34]. Data collection for QUALIDEM outcomes will be conducted separately and will be independent from the use of QUALIDEM as part of the comparison intervention (comparison group 2).

The residents’ challenging behaviors will be assessed with the NPI-NH. This comprehensive instrument includes 12 subscales (delusions, hallucinations, agitation, depression, anxiety, euphoria, apathy, disinhibition, irritability, aberrant motor behavior, night time disturbances, and change in appetite) [33]. Caregivers assess the frequency, severity and the distress caused for each of the 12 domains. Scores can be calculated for each domain (frequency x severity), and those scores can be added together to arrive at a composite score. Total scores range from 0 to 144, and higher scores indicate the presence of more challenging behaviors.

The Physical Self-Maintenance Scale (PSMS) [35] will be used to measure activities of daily living. Dementia severity will be rated using the Functional Assessment Staging (FAST) [36]. Data about regularly prescribed medications will be collected from the residents’ medication documentation sheets. A formal diagnosis of dementia or Parkinson’s disease, as diagnosed by an expert assessment performed by a general practitioner or medical specialist, will be collected from the medical records. Sociodemographic data, including age, gender, and care dependency level as defined by German long-term care insurance, will be collected from the residents’ charts. To ensure standardization, the data collection will always be initiated by trained external research assistants (registered nurses and students in health care study programs).

#### Measures for caregivers

For staff, the primary outcome will be scores obtained from the Attitudes to Dementia Questionnaire (ADQ) [37]. This questionnaire includes 19 items divided into 2 subscales: hope and person-centeredness. Items are summed up to a total score ranging from 19 to 95. The hope subscore includes 8 items with scores ranging from 8 to 40, and the person-centeredness subscore includes 11 items with scores ranging from 11 to 55. Higher scores indicate more positive attitudes toward people with dementia.

Secondary outcome measures include job satisfaction and degree of burnout. Job satisfaction will be measured using the corresponding 4-item scale from the Copenhagen Psychosocial Questionnaire (COSOQ) [38]. The scale calculates a total score ranging from 1 to 4. Higher scores refer to a higher job satisfaction. The degree of burnout will be measured using a 6-item scale from the Copenhagen Burnout Inventory (CBI). The 5 response options for each item result in a total score ranging from 1 to 5. Higher scores correspond to a greater degree of burnout [39]. Both job satisfaction and burnout scales showed an appropriate internal consistency [40].

The dementia-specific burden on staff will be assessed with the German BelaDem Questionnaire [41]. This questionnaire consists of 16 items that rate dementia-specific burden in relation to the challenging behaviors of the residents. Each item will be rated by frequency (rarely, sometimes, often) and severity (not at all, slightly, strong, very strong) over the previous month. Scores can be calculated for each item (frequency x severity), each range from 1 to 12 with higher scores indicating a higher burden. This instrument will be tested for its psychometric properties during the study.

Sociodemographic characteristics (age, gender, working experience), the amount of dementia-specific knowledge, emotional job satisfaction and opportunities to share challenges and opportunities will be assessed with single items.

#### Institution-level measures

Potential institutional influences will be assessed in relation to the organizational characteristics of the respective unit and the “dementia-friendliness” of the environment. To accomplish this, 2 instruments were developed during the preparation phase of the Leben-QD II study. The Dementia-Institution-Questionnaire (DIQ), which evaluates organizational characteristics, is divided into 2 domains. The first domain consists of 4 items and evaluates whole institution structure and financing. The second domain focuses on the nursing unit and is divided into the resident-specific factors (3 items), structure (1 item) and staffing, including staff turnover (5 items). The DIQ will be completed every 3 months by the study coordinator of the corresponding institution.

To assess “dementia-friendliness” the Dementia Milieu Assessment (DMA) will be used, which consists of 29 items. The DMA is a standardized observation instrument. The factors are divided into an environmental domain (21 items) and a psychosocial domain (8 items). Each item has yes or no dichotomous response options. Scores range from 0 to 21 for the environmental domain and from 0 to 8 for the psychosocial domain. Higher scores indicate a more dementia-friendly environment. The DMA is used during a 2-hour period of observation from 3–5 pm in each facility. The observation is carried out in the public space only, and resident rooms are not included. A scientific staff member with experience in dementia-specific observations is responsible for completing the DMA. Both instruments will be tested for their psychometric properties during the study.

### Economic evaluation

The cost-effectiveness of DCM will be investigated by comparing the intervention group with comparison group 2. Data will be extracted from the nursing home administration system. Intervention costs, including costs for the DCM training, will be estimated. Costs that would not occur in routine practice will not be considered in the investigation (i.e., study-specific costs).

### Process evaluation

Data will be collected continuously throughout the study to investigate intervention implementation in the 3 groups and facilitators of and barriers to implementation.

#### Degree of implementation

Semi-structured interviews with head nurses (T2) and staff nurses (T1) involved in the implementation process in each nursing home will be used to gather information on the degree of implementation following the concept of implementation fidelity [42]. Likewise, single items of the staff questionnaire (T2) will assess implementation fidelity by asking in which way staff members are involved in the intervention and how they judge the implementation process.

Furthermore a random sample of 3 resident records per participating institution (T0, T1, T2) will be collected and reviewed for indicators of successful implementation of PCC. An increase in written statements about psychosocial needs of an individual resident will reflect implementation of PCC.

Environmental assessment using the DMA (T0, T2) will also serve to assess changes during implementation and the degree of implementation. Finally, documents will be collected to reconstruct the implementation process within each institution to see if the implementation process has been conducted as intended. Such documents might include implementation concepts, information sheets/flyers, reports on relevant specific meetings and project-related emails.

#### Factors that influence implementation

The Consolidated Framework for Implementation Research (CFIR) is a theoretical framework for the collection and analysis of potential factors within the Leben QD II study that influence implementation [43]. The CFIR consolidates constructs of published implementation theories, using the question “what works where and why across multiple contexts” [43]. The constructs are sorted into 5 domains: “intervention characteristics,” “outer setting,” “inner setting,” “characteristics of the individuals involved,” and the “process of implementation.” Damschroder LJ, 2009 [43] Different types of data will be collected to explore facilitators of and barriers to DCM implementation, using the CFIR framework.

To explore all CFIR domains, semi-structured interviews will be performed with head nurses (T0, T2) and staff nurses (T1) from each participating nursing home involved in the implementation process. The process documents will provide information about the implementation process within each setting.

Feedback meetings on the DCM process and case conferences in the second comparison group will be performed once per intervention cycle to assess team interaction and the “inner setting.” A German version of the SYMLOG-Rating Scale by Schneider/Orlik [44] and field notes will be used to document these interactions. Finally, data from the questionnaire on organizational characteristics (‘inner setting’) and the staff questionnaire (T0/T2) (‘characteristics of individuals’) will also be used to evaluate the implementation process.

## Data analysis

### Effect analysis

Resident and staff outcomes from the intervention and comparison groups will be compared using descriptive and advanced statistical methods at T0, T1 and T2. A multilevel modeling approach will be conducted to evaluate differences between the intervention group and both control arms regarding the development of primary and all secondary outcomes during the study period. This approach will be used to account for the hierarchical structure of the data (i.e. residents with dementia/staff members are nested within institutions and measurement points). To evaluate the effect of assignment the intention to treat principle (ITT) will be applied. The results of ITT will be compared to a per protocol analysis to assess the effect of intervention adherence (degree of implementation). In all analytical steps, the effect estimates will be adjusted for baseline differences and differences in the influencing factors at all measurement points. We will use subgroup analysis to compare the intervention group to comparison group 2. Residents who drop out of the study or are lost to follow up will be described. A statistician who is blinded to group assignment will perform the statistical analyses.

### Process analysis

For process evaluation, the different data sets will be analyzed using a mixed methods approach [45]. Interviews will be analyzed using qualitative content analysis [46] based on themes and structure of the CFIR [43]. Resident records will also be analyzed with qualitative content analysis, but the records will be evaluated using the DCM framework of psychosocial needs [11] to evaluate the implementation of PCC. Process documents will be analyzed using an Access 2007 database to reconstruct the implementation process. The database will allow frequency and content analysis. Quantitative data sets, including staff questionnaire, structural data, and environmental data, will be analyzed using descriptive statistics. SYMLOG data will be visualized in terms of field diagrams according to the instructions of Bales and Cohen [44]. To integrate quantitative and qualitative data sets, a side-by-side comparison for merged data analysis [45] will be performed per institution. Then, the data will be summarized for the 3 study groups accounting for the influencing factors and the degree of implementation. Quantitative and qualitative results will be discussed together and used to confirm or disconfirm each other. Furthermore, the barriers and facilitators of implementation will be merged in an explanatory way to the results on the degree of implementation.

The final step of data analyses will be the merging of outcome data and process evaluation. To avoid bias in interpretation, effect data and data for process evaluation will be analyzed separately by different researchers.

### Economic analysis

Cost-effectiveness analyses focus on the hypothesized additional cost of DCM in comparison to Qol-Assessment using the QUALIDEM. The cost calculation will be performed from a societal point-of-view. Based on the mentioned outcomes, 2 different incremental cost-effectiveness ratios (ICERs) will be calculated: cost per increase in quality of life scores of the residents and cost per increase in attitudes toward dementia scores. Other outcome measures such as challenging behavior, job satisfaction, and burnout will be financially valued and computed on the cost side of the ICER.

## Study progress

The study design and protocol was approved by the Ethics Committee of the German Society of Nursing Science in August 2010. The collection of informed consent started in April 2011 and was followed by an informational kick-off event for the participating institutions. The assessment of baseline data occurred from July to September 2011. The last follow up measurements, effect and process evaluation, are scheduled for January to April 2013. Analysis of the data and dissemination of results are planned for autumn of 2013.

## Discussion

This quasi-experimental study will provide important information on the effects of DCM on residents and staff, implementation of DCM and cost-effectiveness of DCM in German nursing homes. The findings will be an important basis for the design of a subsequent randomized controlled trial.

DCM is a specific intervention with fixed elements and a defined implementation process. It is known that the implementation of DCM in an institution has a strong influence on residents and staff members, the health care team as a whole and the climate and management of an organization. The effects of DCM are strongly influenced by the interaction between the different levels of the organization and organizational readiness [43]. For example, an interested team with motivated change-agents but with an adverse management might not be able to implement DCM to a degree that will result in an improvement of the Qol of the residents. Thus, in addition to the measurement of the intervention’s effects, a major focus of this study is the evaluation of the degree of implementation and the identification of main barriers to and facilitators of implementation. These findings will help nursing home decision-makers and managers decide whether to use DCM, and the results will help to design an adequate implementation process for DCM and other psychosocial interventions.

The following information will be generated and used in the design of a subsequent RCT on effectiveness of DCM in German nursing homes:

a. The appropriateness of the primary and secondary outcomes and important variables and their measurement.

b. Identification of characteristics of nursing homes and units that have a strong influence on the successful implementation of DCM. This information could be used to define inclusion criteria for nursing homes and units and to ensure maximum organizational readiness.

c. Information about helpful adaptations for the DCM implementation process.

d. Information about the treatment and control differences, attrition rate, standard deviation and the interclass correlation coefficient that is needed for a sample size calculation in a subsequent randomized controlled trial.

e. The subgroup analysis of control residents will give some indications about the spillover-effect of PCC on other residents who were not included in the DCM-cycle.

Overall, this study will provide novel data about the effectiveness and implementation process of DCM in German nursing homes. The study results will provide important information for the national discussion about the improvement of dementia-specific Qol, quality of care in nursing homes and the allocation of resources. The study will be important for decision-making and the design and implementation of complex psychosocial interventions such as DCM. Program implementation plans in nursing homes are often made on the basis of individual experience due to the absence of a body of knowledge about successful implementation strategies. Although our study has some design flaws, including a lack of randomization, it is the most feasible way to obtain firsthand data about the effectiveness and the implementation of DCM in German nursing homes.

## Competing interests

The study is partially funded by the Public Welfare Foundation North Rhine-Westphalia (Stiftung Wohlfahrtspflege Nordrhein-Westfalen) and the Knights of St. John of Jerusalem Nursing Home Section West (Johanniter Seniorenhäuser GmbH, Regionalzentrum West). The Knights of St. John of Jerusalem Nursing Home Section West is also the provider that operates the institutions included in the study. The Knights of St. John of Jerusalem Nursing Home Section West is a well-established welfare organization with a charitable status. The Public Welfare Foundation North Rhine-Westphalia is a public foundation which supports projects in the field of social welfare with public funding. Both organizations have no competing interests. All authors are employees of DZNE and declare that they have no competing interests. Christine Riesner is Strategic Lead for Dementia Care Mapping in Germany. In this function, neither she nor the German Strategic Partner of the Bradford Dementia Group has any influence on planning or conducting the study.

## Authors’ contributions

MH, SB, CR, MND and TQ designed the study. MH and MND wrote the first draft of the manuscript and were responsible for revision. CR and TQ helped draft the manuscript. SB commented on the manuscript. All authors have read and approved the final manuscript.

## Pre-publication history

The pre-publication history for this paper can be accessed here:

http://www.biomedcentral.com/1471-2318/13/53/prepub
